# Oxidative Stress Induced Inflammation Initiates Functional Decline of Tear Production

**DOI:** 10.1371/journal.pone.0045805

**Published:** 2012-10-05

**Authors:** Yuichi Uchino, Tetsuya Kawakita, Masaki Miyazawa, Takamasa Ishii, Hiromi Onouchi, Kayo Yasuda, Yoko Ogawa, Shigeto Shimmura, Naoaki Ishii, Kazuo Tsubota

**Affiliations:** 1 Department of Ophthalmology, Keio University School of Medicine, Tokyo, Japan; 2 Department of Molecular Life Science, Tokai University School of Medicine, Kanagawa, Japan; Klinikum rechts der Isar der TU München, Germany

## Abstract

Oxidative damage and inflammation are proposed to be involved in an age-related functional decline of exocrine glands. However, the molecular mechanism of how oxidative stress affects the secretory function of exocrine glands is unclear. We developed a novel *mev-1* conditional transgenic mouse model (*Tet-mev-1*) using a modified tetracycline system (Tet-On/Off system). This mouse model demonstrated decreased tear production with morphological changes including leukocytic infiltration and fibrosis. We found that the *mev-1* gene encodes Cyt-1, which is the cytochrome b_560_ large subunit of succinate-ubiquinone oxidoreductase in complex II of mitochondria (homologous to succinate dehydrogenase C subunit (SDHC) in humans). The *mev-1* gene induced excessive oxidative stress associated with ocular surface epithelial damage and a decrease in protein and aqueous secretory function. This new model provides evidence that mitochondrial oxidative damage in the lacrimal gland induces lacrimal dysfunction resulting in dry eye disease. Tear volume in *Tet-mev-1* mice was lower than in wild type mice and histopathological analyses showed the hallmarks of lacrimal gland inflammation by intense mononuclear leukocytic infiltration and fibrosis in the lacrimal gland of *Tet-mev-1* mice. These findings strongly suggest that oxidative stress can be a causative factor for the development of dry eye disease.

## Introduction

Dry eye disease is a deficiency in tear instability, mainly induced by low tear production, and a functional decline of the lacrimal gland induced by age-related chronic inflammation [1]–[3]. Such age-related chronic inflammation supported the reported prevalence of dry eye disease [4]–[8]. However, the molecular mechanism of age-related lacrimal gland inflammation is unclear. The main cause of chronic inflammation is postulated to involve oxidative stress, and the main endogenous source of oxidative stress is the electron transport chain in mitochondria [9]. The *mev-1* mutant of the nematode *Caenorhabditis elegans* has a genetic dysfunction in complex II of the mitochondrial electron transport chain [10] and overproduces a superoxide anion (O_2_^−^) from the mitochondria [11]. The lifespan of this *mev-1* mutant decreases dramatically as oxygen concentrations are increased from 1 to 60% [12]. In addition, *mev-1*-like dominant negative SdhC (SdhC^171E^) increases oxidative stress and reduces the lifespan in *Drosophila* [13].

To determine whether mouse lacrimal gland functional decline is related to oxidative-stress-induced inflammation, a *mev-1* conditional transgenic mouse (*Tet-mev-1*) was established with a modified tetracycline system (Tet-On/Off system) [14], which equilibrates transgene expression to endogenous levels [15]. Excessive oxidative stress induces mitochondrial respiratory chain dysfunction and results in excessive apoptosis leading to low birth weight and growth retardation in *Tet-mev-1* mice [14]. Using this mouse model, we found that the lacrimal gland of *Tet-mev-1* mice produced more O_2_^−^ and oxidative protein than the lacrimal gland of wild type mice. This new model provides evidence that mitochondrial oxidative damage in the lacrimal gland induces lacrimal dysfunction resulting in dry eye disease.

## Methods

### Animals and Materials

C57BL/6L and *Tet-mev-1* mice were bred and maintained under specially pathogen free (SPF) conditions in the Center of Genetic Engineering for Human Disease (CGHED) (Tokai University School of Medicine, Kanagawa, Japan). Doxycycline was administered in a drinking water mix (dose: 2 mg/ml). All mice used in analyses were 3 month old males.

### Histopathology

Under the operating microscope, the lacrimal gland and submandibular salivary gland were surgically excised after death. A portion of each dissected specimen was immediately embedded in optimal cutting temperature (OCT) compound (Tissue-Tek; Miles Inc., Elkhart, IN, USA) and snap frozen in pre-cooled isopentane at −80°C. The remainder of the tissues was analyzed after being fixed in 4% paraformaldehyde or 10% neutral buffered formalin and embedded in paraffin wax.

#### HE staining and Azan staining

Five micrometer-thick paraffin embedded sections fixed in 4% paraformaldehyde were cut and stained with HE. Additionally, 5 µm-thick paraffin embedded sections fixed in 10% neutral buffered formalin underwent Azan staining to evaluate the severity of fibrosis in the lacrimal gland.

#### Immunohistochemical analysis of DNA damage due to oxidative stress (8-OHdG)

The 5 µm-thick paraffin embedded sections fixed in 4% paraformaldehyde were cut and stained with a mouse anti-8-OHdG monoclonal antibody (Japan Institute for the Control of Aging [JaICA], Shizuoka, Japan) to analyze DNA damage due to oxidative stress [16], [17]. After removal of paraffin, the sections were placed in 10 mM citrate buffer solution and autoclaved at 121°C for 10 min. After blocking with 10% normal goat serum (Vector Laboratories, Burlingame, CA), sections were first blocked with Avidin/Biotin blocking reagent (Vector Labs) and then with a mouse on mouse blocking reagent (M.O.M.™). Blocking with the anti-mouse IgG blocking reagent (Vector Laboratories) was completed overnight at 4°C. Sections were exposed to diluted mouse anti-8-OHdG monoclonal antibody (1∶10). Antibody binding was detected with a horse anti-mouse IgG ABC kit (Vector Laboratories) according to the manufacturer's protocol. The bound antibodies were visualized by the addition of diaminobenzidine tetrahydroxychloride.

#### Analysis of the mononuclear cell fraction using histochemical staining (CD4, CD8, CD19 and F4/80)

Immunohistochemical analysis was performed according to a standard protocol with a panel of mouse monoclonal antibodies specific for CD4, CD8, CD19 and F4/80, (eBioscience, San Jose, CA) [18], [19]. Briefly, 8 µm-thick frozen sections were air dried, fixed in acetone for 20 min at room temperature, and rehydrated in phosphate-buffered saline (PBS). Nonspecific binding was inhibited by incubating the specimens with 5% goat serum in PBS for 30 min at room temperature. The sections were incubated with the optimally diluted primary antibody at room temperature for 2 h, followed by incubation with a peroxidase-conjugated rabbit anti-mouse IgG antibody (Histofine® Simple Stain Rat MAX PO (M)) (Nichirei Biosciences Inc, Tokyo, Japan) for 45 min. The bound antibodies were visualized by the addition of diaminobenzidine tetrahydroxychloride. All steps were followed by three washes with PBS. Nuclei were counterstained with hematoxylin for 1 min [20].

### Quantitative real-time RT-PCR

#### RNA extraction

An acid guanidinium-phenol-chloroform method was used to isolate RNA from tissues and cultured cells. The following protocol describes isolation of RNA from mouse lacrimal gland tissue. Immediately after removal from the animal, the tissue was minced on ice and homogenized (at room temperature) with 0.85 ml of 4 M guanidinium thiocyanate (GTC) in a glass-Teflon homogenizer and subsequently transferred to a 15 ml polypropylene tube with 2 ml of 4 M GTC, 0.15 ml of 10% sarcosyl and 0.72 µl of 2-mercaptoethanol. A total of 0.3 ml of 2 M sodium acetate, pH 4, 3 ml of phenol (water saturated), and 0.6 ml of chloroform-isoamyl alcohol mixture (24∶l) were sequentially added to the homogenate, with thorough mixing by inversion after the addition of each reagent. The final suspension was shaken vigorously for 10 s and cooled on ice for 15 min. Samples were centrifuged at 7000 rpm for 20 min at 4°C. After centrifugation, RNA was present in the aqueous phase whereas DNA and proteins were present in the interphase and phenol phase. The aqueous phase was transferred to a fresh tube, mixed with 3 ml of isopropanol, and then placed at −20°C for at least 2 h to precipitate the RNA. Centrifugation at 7000 rpm for 20 min at 4°C was again performed and the resulting RNA pellet was washed in 3 ml of 70% ethanol and centrifuged at 7000 rpm for 20 min at 4°C. After centrifugation, the RNA pellet was air-dried (1 h) at room temperature. After drying, 88 µl 0.1% diethyl pyrocarbonate (DEPC) in distilled water was added to the pellet. The solution was transferred to a 2 ml Eppendorf tube with 2 µl DNase (20 U), 10 µl DNase buffer and 0.5 µl RNase inhibitor (Pharmacia) and was heated for 30 min at 37°C. After cooling on ice, the solution was added to 400 µl of a chloroform-phenol mixture (1∶l) and 300 µl of 0.1% DEPC in distilled water. After 20 min on ice, the solution was centrifuged at 12000 rpm for 20 min at 4°C. The aqueous phase was transferred to a fresh tube with 35 µl 3 M sodium acetate and 1 ml 100% ethanol. After mixing, this solution was placed at −20°C for 30 min and centrifuged at 12000 rpm for 20 min at 4°C. The sediment was washed with 400 µl 70% ethanol and centrifuged at 12000 rpm for 5 min at 4°C. The sediment was air-dried for 1 h at room temperature and 100 µl 0.1% DEPC in distilled water was added.

#### Complementary DNA (cDNA) preparation and quantitative real-time RT-PCR

First strand complementary DNA (cDNA) was synthesized from 4.0 µg of total RNA using SuperScript III Reverse Transcriptase (Invitrogen) according to the manufacturer's protocol. RT-PCR primers and an appropriate probe were chosen by the Universal Probe Library (UPL) Assay Design Center web service. Quantitative real-time RT-PCR was performed with pre-designed primers (Nihon Gene Research Laboratories, Sendai, Japan) and a TaqMan® probe (Applied Biosystems, Foster City, CA, USA) for the housekeeping gene GAPDH (NM 008084.2) (forward primer [FP]: AGCTTGTCATCAACGGGAAG, reverse primer [RP]: TTTGATGTTAGTGGGGTCTCG) (UPL probe: #9) as an endogenous control to normalize the expression data for each gene: IL-1β (NM 008361.3) (FP:TGTAATGAAAGACGGCACACC, RP:TCTTCTTTGGGTATTGCTTGG) (UPL probe #78), tumor necrosis factor (TNF-α) (NM 013693.2) (FP:TGCCTATGTCTCAGCCTCTTC, RP:GAGGCCATTTGGGAACTTCT) (UPL probe #49), IL-6 (NM 031168.1) (FP:GCTACCAAACTGGATATAATCAGGA,RP:CCAGGTAGCTATGGTACTCCAGAA) (UPL probe #6), IL-10 (NM 010548.1) (FP:CAGAGCCACATGCTCCTAGA,RP:TGTCCAGCTGGTCCTTTGTT) (UPL probe #41) and interferon-γ (IFN-γ) (NM 008337.3) (FP:ATCTGGAGGAACTGGCAAAA, RP:TTCAAGACTTCAAAGAGTCTGAGGTA) (UPL probe #21). Quantitative real-time RT-PCR was completed using the TaqMan® Gene Expression Assay and the Applied Biosystems 7500 Real-time PCR system (Applied Biosystems).

### Isolation of mitochondria

Mitochondria were isolated from mouse lacrimal glands using a standard procedure involving differential centrifugation [21], [22]. After washing with ice-cold PBS, the lacrimal glands were minced in a volume of isolation buffer (210 mM mannitol, 70 mM sucrose, 0.1 mM EDTA, and 5 mM Tris-HCl, pH 7.4). The minced lacrimal glands were homogenized in isolation buffer at 800 rpm with 30 strokes using a Teflon homogenizer. The homogenate was centrifuged at 2000 rpm for 10 min at 4°C. The supernatant was transferred to a fresh tube and centrifuged at 14000 rpm for 10 min at 4°C. The mitochondria-containing pellet was suspended in TE buffer (50 mM Tris-HCl pH 7.4 and 0.1 mM EDTA).

### Measurement of activity of complexes I and II of the electron transport chain

The activity of NADH-coenzyme Q oxidoreductase (complex I) and succinate-coenzyme Q oxidoreductase (complex II) in mitochondria was measured as previously described [22], [23]. Tissues were homogenized in isolation buffer (10 mM HEPES, pH 7.4, 0.15 M NaCl). The resulting homogenate was centrifuged at 250× g for 10 min to remove debris. The supernatant was further centrifuged at 31000× g for 20 min. The pellet was suspended in isolation buffer. Complex I activity was assayed by measuring NADH-sensitive NADH-cytochrome *c* reductase activity at 37°C in 200 µl 0.1 M Tris–SO_4_ buffer at pH 7.4, containing 0.32 mg cytochrome *c* and 1 mM sodium cyanate. Complex II activity was assayed by measuring malonate-sensitive succinate-cytochrome *c* reductase activity. The reference cuvette contained 20 µl of 20% sodium malonate solution.

### Measurement of O_2_ *^−^*

Production of O_2_ *^−^* was measured using the chemiluminescent probe 2-methyl-6-p-methoxyphenylethynyl-imidazopyrazinone (MPEC) (ATTO Co., Tokyo, Japan). MPEC has an advantage of low background relative to 3, 7-dihydro-2-methyl-6-(4-methoxyphenol) imidazole [1, 2-a] pyrazin-3-one (MCLA), which is generally used [15], [23]–[25]. A total of 40 µg of intact mitochondrial fraction was added to 1 ml assay buffer (50 mM HEPES-NaOH, pH 7.4 and 2 mM EDTA) containing 0.7 µM of MPEC. The solutions were placed in a photon counter with an AB-2200 type Luminescencer-PSN (ATTO Co.) and measured at 37°C. The rates of O_2_ *^−^* production were expressed as counts per second.

### Measurement of carbonylated protein

Carbonylated protein as an indicator of oxidized protein was detected by an enzyme linked immunosorbent assay (ELISA) [25]. Isolated mitochondrial proteins from the lacrimal gland were treated with 10 mM DNPH. A total of 250 ng of mitochondrial protein in 50 mM NaHCO_3_ was coated on an enhanced protein-binding ELISA plate (Caster) by incubating at 4°C for 8 h. Nonspecific binding to the plate was minimized by blocking the wells with 100 µl blocking buffer (3% BSA and 0.1% NaN_3_ in PBS) at 37°C for 1 h. After the supernatant was removed, 100 µl of anti-DNP antibody diluted with buffer G (0.1% BSA, 0.1% gelatin, 0.1% NaN_3_ and 1 mM MgCl_2_ in PBS) was added to each well and incubated at 37°C for 1 h. After the supernatant was removed, the plate was washed four times with PBS and 100 µl of horseradish peroxidase-conjugated secondary antibody diluted with 0.05% Tween 20 in PBS was added followed by incubation at 37°C for 1 h. The plate was washed four times to remove the unbound secondary antibody. After 100 µl of ELISA coloring solution (0.0156 M C_6_H_8_O_7_, 0.1 M Na_2_HPO_4_·12H_2_O, 0.4 mg/ml o-phenylenediamine dihydrochloride and 0.2 µl/ml 30% H_2_O_2_) was added to each well, the reaction was terminated by the addition of 100 µl of 1 M H_2_SO_4_. The absorbance was measured using a computer-controlled spectrophotometric plate reader (Spectra Max 250: Molecular Devices) at a wavelength of 492 nm.

### Corneal fluorescein staining

Corneal fluorescein staining was performed as described by Rashid et al. [26]. Sodium fluorescein (1%) was applied to the cornea of mice. Three minutes later, eyes were flushed with PBS to remove excess fluorescein, and corneal staining was evaluated with a hand slit lamp (Kowa, Tokyo, Japan) using cobalt blue light. Punctate staining was recorded using a standardized grading system of 0 to 3 for each of the three areas of the cornea [27]–[29].

### Aqueous tear measurement

For 3 min, tears (0.5 µl) from each mouse were collected in a microcapillary tube. Tear volume was measured using capillary length (mm). Tear volume was normalized against the body weight of each mouse and the experiments were performed three times to validate the tear measurement.

## Results

**Figure 1:**
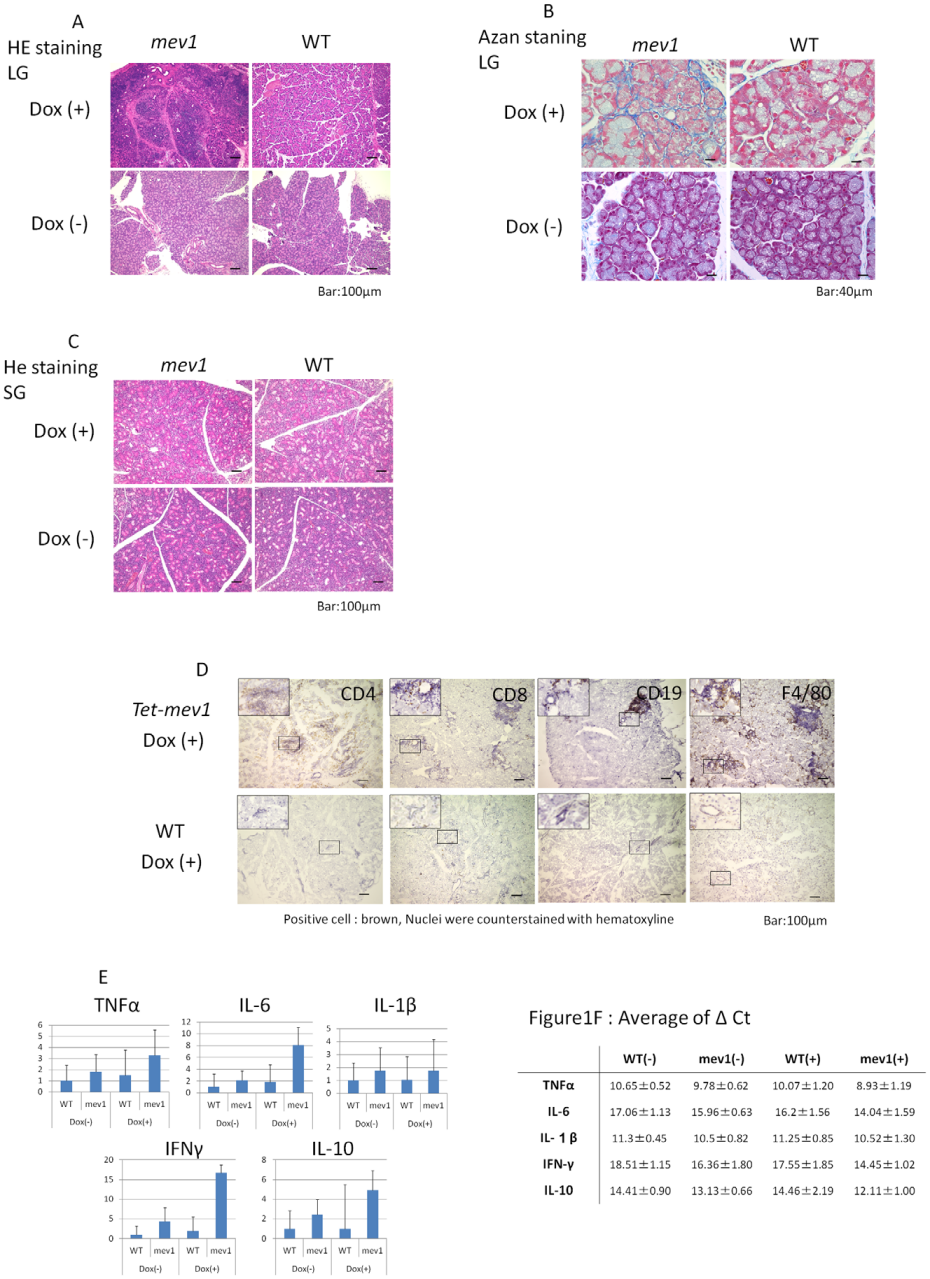
Inflammation of the lacrimal gland in *Tet-mev-1 mice* with Dox. A, HE staining shows that *Tet-mev-1 mice* with Dox (*Tet-mev-1*/Dox(+)) typically have multifocal inflammation. The other types of mice (*Tet-mev-1*/Dox(−), WT/Dox(+) and WT/Dox(−)) have no inflammation in the lacrimal gland. Scale bar, approximately 100 µm. B, Azan staining was used to evaluate the severity of fibrosis in the lacrimal gland. *Tet-mev-1*/Dox(+) only shows fibrosis around acinar cells in the lacrimal gland. Scale bar, approximately 40 µm. C, Histopathology of the salivary glands shows no inflammation in all types of mice. Scale bar, approximately 100 µm. D, In lacrimal glands of *Tet-mev-1*/Dox (+) mice, CD4^+^ T cells, CD8^+^ T cells, CD19^+^ cells (B cells) and F4/80^+^ cells (pan-macrophage) were observed. Scale bar, approximately 100 µm. E, Proinflammatory cytokines were evaluated by real-time RT-PCR (ratio to WT/Dox(−)). Proinflammatory cytokines (TNF-α, IL-6, IL-1β, and IFN-γ) were increased in *Tet-mev-1*/Dox(+), especially IL-6 and IFN-γ, and IL-10 was also increased. F, Row data about Proinflammatory cytokines evaluated by Real-time RT-PCR is shown.

Histopathology of the lacrimal glands revealed no inflammation in *Tet-mev-1* mice without Dox (*Tet-mev-1*/Dox(−)) or in wild type mice (C57BL/6J) with Dox (WT/Dox(+)) or without Dox (WT/Dox(−)) at 3 months old. *Tet-mev-1*/Dox(+) mice typically had multifocal inflammation and fibrosis around acinar cells in the lacrimal gland (Fig. 1a, b). However, histopathology of the salivary glands showed no inflammation in all mice (Fig. 1c). Moreover, although the superoxide anion was overproduced in the whole body of *Tet-mev-1*/Dox(+) mice, other main internal organs examined (i.e., liver, heart, kidney, lung and brain) did not have an inflammatory response (data not shown). To clarify the inflammatory status, we investigated the immunostaining by cell surface antigens (CD4, CD8, CD19, and F4/80). Various immunocytes such as cytotoxic T cell, helper T cells, activated B cells, and pan-macrophages had infiltrated the inflammatory focus (Fig. 1d). This inflammation was not observed in WT/Dox(+) mice, which suggested that doxycycline administration did not cause inflammation in the lacrimal gland. In addition, quantitative real-time RT-PCR analysis of the cytokines in the lacrimal gland showed an increase in inflammatory cytokines including TNFα, IL-6 and INFγ, which may be related to the inflammatory reaction in the lacrimal gland of *Tet-mev-1*/Dox(+) mice. Expression of the anti-inflammatory cytokine IL-10 was increased. (Fig. 1e, f).

**Figure 2:**
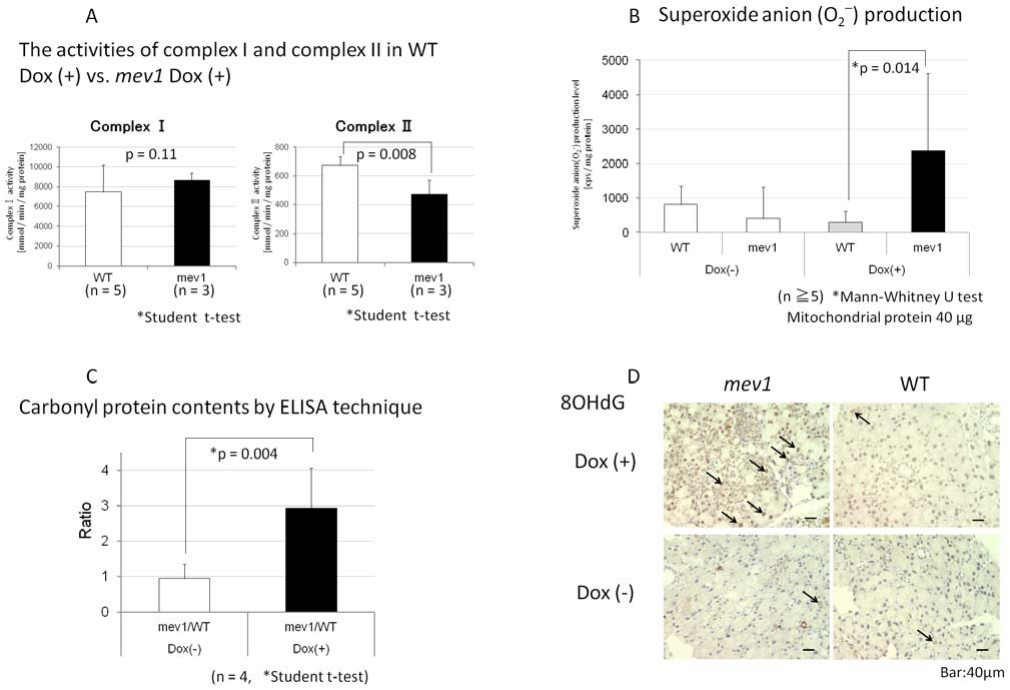
Lacrimal gland in *Tet-mev-1 mice* with Dox has functional depression of mitochondria and excessive O_2_^−^production. A, The activity of complexes I and II in WT/Dox(+) vs. *Tet-mev-1* mice/Dox(+). NADH-cytochrome c oxidoreductase was applied as an enzymatic indicator of complex I activity, and succinate-coenzyme Q oxidoreductase as an enzymatic indicator of complex II activity. Although there were no differences in the activity of complex I between these mice, complex II was significantly decreased in *Tet-mev-1 mice* with Dox. (WT: n = 5, *Tet-mev-1*: n = 3, NS, not significant; *P<0.01 [Student's t-test]). The vertical bars indicate the standard deviation of the separate experiments. B, Production of O_2_^−^ in the lacrimal gland was significantly increased in *Tet-mev-1*/Dox(+) compared with that in the other types of mice. (n≥5, *P = 0.0014 [Kruskal-Wallis test]). The vertical bars indicate the standard deviation of the separate experiments. C, Carbonyl protein content of the lacrimal gland by ELISA. Each value shows the ratio of *Tet-mev-1* and WT for the relative amount of carbonyl protein in *Tet-mev-1 mice* with or without Dox (n = 4, *P = 0.004 [Student's t-test]). D, Immunohistochemical staining of 8-OHdG: *Tet-mev-1*/Dox(+) shows more positive nuclei (brown, indicated by the arrow) than the other types of mice.

*Tet-mev-1* mice contain the mutation site of SDHC V69E, which is located within the functional ubiquinone (CoQ)-binding region of complex II [15], [30], [31]. *Tet-mev-1* mice are conditional transgenic mice and were designed to have decreased affinity of CoQ for complex II in mitochondria, which would induce electron leakage and lead to an increase in production of superoxide anion from complex II in the presence of doxycycline. The activity of complexes I and II in mitochondria of the lacrimal gland was compared between WT/Dox(+) and *Tet-mev-1*/Dox(+) mice. In the mitochondria of the *Tet-mev-1* mouse, only the activity of complex II was decreased, and, thus, reactive oxygen species (ROS) was overproduced from complex II with doxycycline. According to the intended design of the model, complex I activity of the lacrimal gland was not significantly different between WT/Dox(+) and *Tet-mev-1*/Dox(+) mice, and complex II activity in *Tet-mev-1*/Dox(+) mice was significantly lower than in WT/Dox(+) mice (p = 0.008, Fig. 2a). The activity of complex II-induced O_2_^−^ production in the lacrimal gland significantly increased in *Tet-mev-1*/Dox(+) mice compared with that in the other types of mice (p = 0.014, Fig. 2b). We then measured carbonylated protein as a marker of oxidized proteins, which accumulate in the mitochondrial fractions of wild type mice during aging [25]. Our results showed that carbonylated protein amounts in the lacrimal gland of wild type mice were not significantly different between Dox(+) and Dox(−) mice. Therefore, doxycycline did not affect the quantity of carbonylated protein. Carbonylated protein content was determined by ELISA and the ratio of WT/Dox(+) and *Tet-mev-1*/Dox(+) was three times higher than the ratio of WT/Dox(−) and *Tet-mev-1*/Dox(−) (p<0.01, Figure 2c). The compound 8-OHdG accumulates with aging [32], and accordingly, 8-OHdG was used as a marker of oxidative damage in DNA in our study. Immunohistological labeling intensity for 8-OHdG was higher in the lacrimal gland of *Tet-mev-1*/Dox(+) mice compared with that in the other types of mice (Fig. 2d).

**Figure 3:**
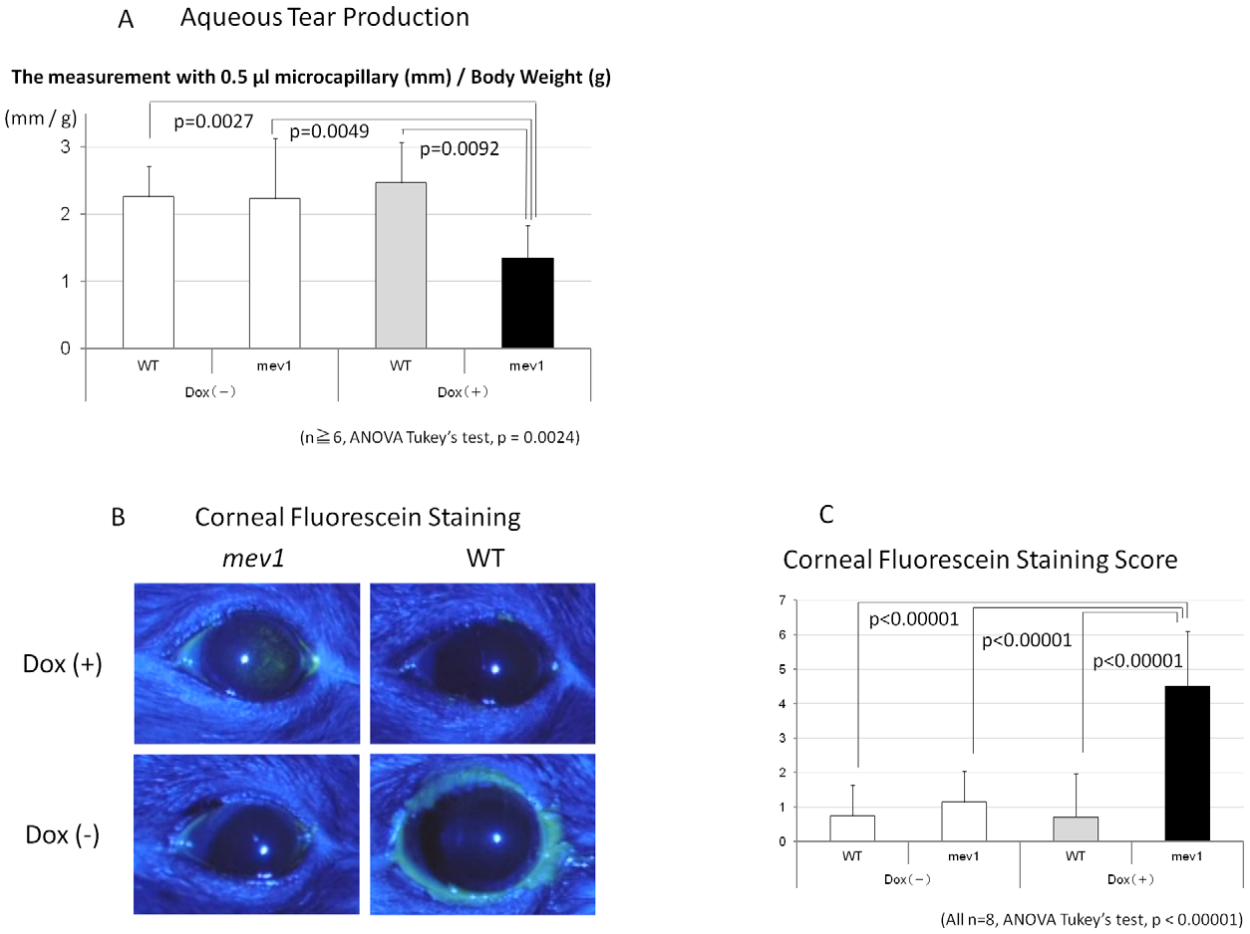
*Tet-mev-1*/Dox(+) have dry eye disease. A, Aqueous tear production: Aqueous tear quantity values of *Tet-mev-1*/Dox(+) were significantly lower than those in the other types of mice (n≥6, ANOVA Tukey's test, p = 0.0024). B, *Tet-mev-1*/Dox(+) mice had more corneal fluorescein staining than in the other mice. C, The corneal fluorescein staining score of *Tet-mev-1*/Dox(+) was significantly worse than that in the other types of mice (all n = 8, ANOVA Tukey's test, p<0.00001).

The aqueous tear quantity values were 2.26±0.48 mm/g (n = 14), 2.23±0.46 mm/g (n = 6), 2.47±0.60 mm/g (n = 6), and 1.35±0.48 mm/g (n = 8) for WT/Dox(−) mice, *Tet-mev-1*/Dox(−) mice, WT/Dox(+) mice, and *Tet-mev-1*/Dox(+) mice, respectively. The aqueous tear quantity values for *Tet-mev-1*/Dox(+) mice were significantly lower than in the other types of mice (n≥6, ANOVA Tukey's test, p = 0.0024) (Fig. 3a). Corneal fluorescein staining was higher in *Tet-mev-1*/Dox(+) mice compared with that in the other three types of mice (Fig. 3b). The corneal fluorescein staining scores were 0.75±0.89, 1.14±0.90, 0.71±1.25, and 4.50±1.60 for WT/Dox(−) mice, *Tet-mev-1*/Dox(−) mice, WT/Dox(+) mice, and *Tet-mev-1*/Dox(+) mice (all n = 8), respectively. The score ranged from 0 to 9 with a score of 0 indicating normal and a score of 9 indicating a severe corneal punctuate defect. The corneal fluorescein staining score for *Tet-mev-1*/Dox(+) mice was significantly worse than for the other three types of mice (n = 8, ANOVA Tukey's test, p<0.00001) (Fig. 3c).

## Discussion

It is well known that lacrimal and salivary gland functions decline with age in humans [33], [34]. We first hypothesized that both lacrimal and salivary gland functions decline in *Tet-mev-1*/Dox(+) mice. However, the severe inflammation and fibrosis associated with functional decline occurred in the lacrimal gland, but not in the salivary gland. We hypothesized that the inherent tissue responses to oxidative stress in the lacrimal and salivary glands are different. Pharmacological cholinergic blockade (subcutaneous injection of scopolamine hydrobromide) inhibits lacrimal gland function. It also stimulates inflammatory cytokine production and lymphocytic infiltration in the lacrimal gland. This systemic cholinergic blockade does not induce a nonspecific inflammation at three sites (conjunctival goblet cells, submandibular glands and small intestine) that receive cholinergic innervations [35]. These results suggest that the lacrimal gland is subject to inflammation by various stimuli in contrast with the salivary gland.

Mitochondria generate ATP through aerobic respiration, whereby glucose, pyruvate, and NADH are oxidized, thus generating ROS as a byproduct. In normal circumstances, the deleterious effects caused by the highly reactive nature of ROS are balanced by the presence of antioxidants. However, high levels of ROS are observed in chronic human diseases such as neurodegeneration [36], digestive organ inflammation [37], and cancer [38]. Recent work exploring the mechanisms linking ROS and inflammation suggest that ROS derived from mitochondria (mtROS) act as signal transducing molecules to trigger pro-inflammatory cytokine production [39]. Cells from patients with TNFR1-associated periodic syndrome (TRAPS) demonstrate that increased mtROS levels influence the transcription of pro-inflammatory cytokines such as IL-6 and TNF. TRAPS manifests as episodes of fever and severe localized inflammation with mutations in TNFR1. Inhibition of mtROS production inhibited MAPK activation and production of IL-6 and TNF in cells from TRAPS patients [40]. The mtROS in *Tet-mev-1*/Dox(+) mice may also directly induce increasing production of TNF-α and IL-6 and continuously induce inflammation in the lacrimal gland.

Protein oxidation is a biomarker of oxidative stress and many different types of protein oxidative modification can be induced directly by ROS or indirectly by reactions of secondary by-products of oxidative stress [41]. Lacrimal gland function has been reported to decrease gradually with aging, leading to reduced tear secretion and dry eye disease in the elderly [3], [7]. Aging occurs, in part, as a result of the accumulation of oxidative stress caused by ROS that are generated continuously during the course of metabolic processes. Levels of 8-OHdG as a DNA oxidative stress marker and 4-HNE as a by-product of lipid peroxidation are higher and tear volume is decreased in middle-aged rats. Caloric restriction prevents a decline in lacrimal gland function and morphological changes and might be associated with a reduction in oxidative stress [42].

We confirmed that 8-OHdG immunohistological labeling intensity was higher in the lacrimal gland of *Tet-mev-1*/Dox(+) mice than in other mice types and the ratio of carbonylated protein content in mice with Dox was three times the ratio of mice without Dox. Collectively, mtROS production may damage DNA and induce the accumulation of carbonylated protein in the lacrimal gland.

These biochemical and histochemical data suggest that overproduced superoxide anion from the mitochondria affect directly and/or indirectly oxidative damage and inflammation in the lacrimal gland. It is believed that chronic inflammation of the lacrimal gland is a major contributor to insufficient tear secretion. Chronic inflammation of the lacrimal gland occurs in several pathologic conditions such as autoimmune diseases (Sjögren syndrome, sarcoidosis, and diabetes) or simply as a result of aging [43]. The relationship between inflammation of the lacrimal gland and tear secretion deficiency has been described [44], [45]. IL-1β induces a severe inflammatory response in the lacrimal gland and inhibits lacrimal gland secretion and subsequent dry eye disease [44]. A single injection of interleukin-1 into the lacrimal glands induces reversible inflammation and leads to destruction of lacrimal gland acinar epithelial cells, which results in decreased tear production. However, these inflammatory responses subside and lacrimal gland secretion and tear production return to normal levels [45].

For the dry eye model, we first reported the accelerated oxidation of protein, lipid, and DNA of the ocular surface in the rat swing model [46], [47]. Accumulated oxidative damage caused the functional decline of the lacrimal gland and dry eye disease in *Tet-mev-1/*Dox(+) mice. In the lacrimal gland, age-related chronic inflammation, and age-related functional alterations including decreased acetylcholine release and protein secretion, might be related to dry eye diseases [48], [49]. Our study clearly demonstrated that oxidative stress from mitochondria induced dry eye disease with morphological changes in the lacrimal gland of mice. In conclusion, reducing oxidative stress might be one of the possible treatments for age-related/ROS-induced dry eye disease.
