# Correction: Oxidative Stress Induced Inflammation Initiates Functional Decline of Tear Production

**DOI:** 10.1371/journal.pone.0127720

**Published:** 2015-05-11

**Authors:** Yuichi Uchino, Tetsuya Kawakita, Masaki Miyazawa, Takamasa Ishii, Hiromi Onouchi, Kayo Yasuda, Yoko Ogawa, Shigeto Shimmura, Naoaki Ishii, Kazuo Tsubota

The authors of this article wish to deeply apologize for the use of verbatim text from previous publications and for the lack of appropriate citation and quotations. The overlap in the text from previous publications relates to portions of the Discussion section of the article, as provided below:

The following portion in the Discussion overlaps with Naik and Dixit (2011). Mitochondrial reactive oxygen species drive proinflammatory cytokine production, J Exp Med 208, 3, 417–420:

“Mitochondria generate ATP through aerobic respiration, whereby glucose, pyruvate, and NADH are oxidized, thus generating ROS as a byproduct. In normal circumstances, the deleterious effects caused by the highly reactive nature of ROS are balanced by the presence of antioxidants. However, high levels of ROS are observed in chronic human diseases such as neurodegeneration [36], digestive organ inflammation [37], and cancer [38]. Recent work exploring the mechanisms linking ROS and inflammation suggest that ROS derived from mitochondria (mtROS) act as signal transducing molecules to trigger pro-inflammatory cytokine production [39]. Cells from patients with TNFR1-associated periodic syndrome (TRAPS) demonstrate that increased mtROS levels influence the transcription of pro-inflammatory cytokines such as IL-6 and TNF. TRAPS manifests as episodes of fever and severe localized inflammation with mutations in TNFR1…Inhibition of mtROS production inhibited MAPK activation and production of IL-6 and TNF in cells from TRAPS patients.”

The following text in the Discussion overlaps with Kawashima et al. (2010), cited at the end of the paragraph:

“Lacrimal gland function has been reported to decrease gradually with aging, leading to reduced tear secretion and dry eye disease in the elderly [3, 7]. Aging occurs, in part, as a result of the accumulation of oxidative stress caused by ROS that are generated continuously during the course of metabolic processes.”

The following text in the Discussion overlaps with Zoukhri et al. (2008). Mechanisms of Murine Lacrimal Gland Repair after Experimentally Induced Inflammation, Invest. Ophthalmol. Vis. Sci. 49, 10, 4399–4406:

“It is believed that chronic inflammation of the lacrimal gland is a major contributor to insufficient tear secretion. Chronic inflammation of the lacrimal gland occurs in several pathologic conditions such as autoimmune diseases (Sjögren syndrome, sarcoidosis, and diabetes) or simply as a result of aging [43].”

The following sentence overlaps with Dalle-Donne et al. (2003). Protein carbonylation in human diseases, Trends in Molecular Medicine, 9, 4, 169–176:

“Protein oxidation is a biomarker of oxidative stress and many different types of protein oxidative modification can be induced directly by ROS or indirectly by reactions of secondary by-products of oxidative stress”

The above was incorrectly cited as Berlett and Stadtman (1997).

The authors declare that the text overlap has no effect on the results and conclusions of the study and apologize for the instances of plagiarism above.

The authors would also like to note that some of the results from this article were also published in the journal Cornea shortly after publication in PLOS ONE. The relevant article in Cornea can be found here: http://journals.lww.com/corneajrnl/Abstract/2012/11001/A_New_Mouse_Model_of_Dry_Eye_Disease___Oxidative.12.aspx

## References

[1] Uchino Y, Kawakita T, Miyazawa M, Ishii T, Onouchi H, Yasuda K, et al (2012) Oxidative Stress Induced Inflammation Initiates Functional Decline of Tear Production. PLoS ONE 7(10): e45805 doi: 10.1371/journal.pone.0045805 23071526 10.1371/journal.pone.0045805 PMC3465290

